# Supplementary material for: Chromothripsis during telomere crisis is independent of NHEJ, and consistent with a replicative origin
Source: Genome Res. 2019 May;29(5):737–49. doi: 10.1101/gr.240705.118 (PMC6499312; doi:10.1101/gr.240705.118)
Supplement: Supplemental Material [file supp_gr.240705.118_Supplemental_file_1.zip › contigs/annotated_contigs/DB50/contig.2.DB50_length_481_mean_cov_11.7546777547.docx]

**DB50_length_481_mean_cov_11.7546777547**

```
AGGCCTTTAAATGTGCCAACTCAAGGAGCCTTGGTTGCCCCCTCAGGAAGGGTGCTGGTTGGGGAATTTCAAGGATTGTGTGAGAGGGT
 >chr11:72356359-72356493 - E=5e-69
TTTTCTGAAAGGGCTCTGCACTCTACCAAGCACTGGAAGAAA|GCA|TGGGCAAAGGCCTGGAGGTGAGAGTCAGTCAGTCATTGATGT
                                           >chr11:72354773-72355123 - E=3e-196
GAGAAGAGCAAGAAGTAGAAATGTAAGGAATGGTGGGGAGGGGAGTCAGAGCTGGATGACCAAGCAAGGGTTCAGCTGTAGAGGGTCTG

GCCCGCCAGGCTCAGGGCTCGGGCTTTATTGTGCTGGTGGTAGGGAGCCACTGAGGGTGAGTGGGGGAGAGCATGCCAGAGCATGCCTC

AGAAAGAAAGGTGGGAGAAACGCTGGCATGGAGGGCCGCCCCCTGAGTTGGTGGGGTGGCCGGGCTCTGCCAAGGCTATGTGCCAGCTG

CCTGGACTGTGTCCAGGAATGGGCACAATGACTCAACA
```
